# Author Correction: Stroke subtype-dependent synapse elimination by reactive gliosis in mice

**DOI:** 10.1038/s41467-022-28885-6

**Published:** 2022-02-28

**Authors:** Xiaojing Shi, Longlong Luo, Jixian Wang, Hui Shen, Yongfang Li, Muyassar Mamtilahun, Chang Liu, Rubing Shi, Joon-Hyuk Lee, Hengli Tian, Zhijun Zhang, Yongting Wang, Won-Suk Chung, Yaohui Tang, Guo-Yuan Yang

**Affiliations:** 1 School of Biomedical Engineering and Affiliated Sixth People’s Hospital, Shanghai Jiao Tong University, Shanghai, 200030 China; 2 Department of Rehabilitation, Ruijin Hospital, School of Medicine, Shanghai Jiao Tong University, Shanghai, 200025 China; 3 Department of Biological Sciences, Korea Advanced Institute of Science and Technology, Daejeon, 34141 South Korea; 4 Department of Neurology, Ruijin Hospital, School of Medicine, Shanghai Jiao Tong University, Shanghai, 200025 China; 5 Present Address: Dermatology and Venerology Unit, Department of Medicine, Karolinska Institute, Stockholm, Sweden

**Keywords:** Stroke, Glial biology, Cellular neuroscience

Correction to: *Nature Communications* 10.1038/s41467-021-27248-x, published online 26 November 2021.

The original version of this Article contained errors in the labelling of Fig. 1a and Fig. 1g, in which the labels D1, D3, D7 and D14 were inadvertently shifted to the left resulting in the labels D7 and D14 not being aligned with their respective images. This has been corrected in both the PDF and HTML versions of the Article.

The original Article also contained errors in Fig. 5e, 5g, 5p and 5r, in which the labels for Western blot samples were incorrect. In Fig, 5e and 5p sample lanes 4–6 were incorrectly labelled with MERKTK^WT^ and sample lanes 7-9 were incorrectly labelled with C-MEGF10^KO^. In the correct version sample lanes 4–6 are labelled with C-MEGF10^KO^ and sample lanes 7–9 are labelled with MERKTK^WT^. In Fig. 5g and 5r sample lanes 4–6 were incorrectly labelled with MERTK^WT^ and sample lanes 7–9 were incorrectly labelled A-MEGF10^KO^.

In the correct version sample lanes 4–6 are labelled with A-MEGF10 ^KO^ and sample lanes 7–9 are labelled with MERTK^WT^. These have been corrected in both the PDF and HTML versions of the article. The same labelling errors in Fig. 5e, 5g, 5p and 5r were also present in Supplementary Fig. 12, which contained the uncropped Western blots corresponding to Fig 5e, 5g, 5p and 5r. The HTML has been updated to include a corrected version of the Supplementary information file.

## Supplementary information

Supplementary Figures

