# Supplementary material for: Stroke subtype-dependent synapse elimination by reactive gliosis in mice
Source: Nat Commun. 2021 Nov 26;12:6943. doi: 10.1038/s41467-021-27248-x (PMC8626497; doi:10.1038/s41467-021-27248-x)
Supplement: Supplementary file 2 — Reporting Summary [file 41467_2021_27248_MOESM2_ESM.pdf]

nature research

Corresponding author(s): Guo-Yuan Yang ; Yaohui Tang ; Won-Suk Chung

Last updated by author(s): Nov 4, 2021

Reporting Summary

Nature Research wishes to improve the reproducibility of the work that we publish. This form provides structure for consistency and transparency in reporting. For further information on Nature Research policies, see our Editorial Policies and the Editorial Policy Checklist.

Statistics

For all statistical analyses, confirm that the following items are present in the figure legend, table legend, main text, or Methods section.

n/a	Confirmed	
☐	☒	The exact sample size (n) for each experimental group/condition, given as a discrete number and unit of measurement
☐	☒	A statement on whether measurements were taken from distinct samples or whether the same sample was measured repeatedly
☐	☒	The statistical test(s) used AND whether they are one- or two-sided<br>*Only common tests should be described solely by name; describe more complex techniques in the Methods section.*
☒	☐	A description of all covariates tested
☒	☐	A description of any assumptions or corrections, such as tests of normality and adjustment for multiple comparisons
☐	☒	A full description of the statistical parameters including central tendency (e.g. means) or other basic estimates (e.g. regression coefficient) AND variation (e.g. standard deviation) or associated estimates of uncertainty (e.g. confidence intervals)
☐	☒	For null hypothesis testing, the test statistic (e.g. F, t, r) with confidence intervals, effect sizes, degrees of freedom and P value noted<br>*Give P values as exact values whenever suitable.*
☒	☐	For Bayesian analysis, information on the choice of priors and Markov chain Monte Carlo settings
☒	☐	For hierarchical and complex designs, identification of the appropriate level for tests and full reporting of outcomes
☒	☐	Estimates of effect sizes (e.g. Cohen's d, Pearson's r), indicating how they were calculated

Our web collection on statistics for biologists contains articles on many of the points above.

Software and code

Policy information about availability of computer code

Data collection	Bright-field and fluorescent signals were detected by confocal laser scanning fluorescent microscopy (Leica, Wetzlar, Germany). Western blot results were collected using chemiluminescent imaging system (Tanon 5200). TEM images were imaged under biological transmission electron microscope (Tecnai G2 spirit Biotwin). Single cell RNA samples were sequenced on a HiSeq X Ten platform (Illumina).
Data analysis	Immunohistochemistry signals were quantified using LAS AF Lite version 4.0 (Leica Biosystems, Germany) and ImageJ version 1.51j8 (NIH, Bethesda, MD). 3D images were reconstructed using Imaris version 9.0.1. Western blot results were quantified using ImageJ software. Bright field image and chemiluminescent blots are merged using Tanon GIS software Version 1.0. The length and number of dendrite spine were calculated using Reconstruct version 1.1.0.0. The statistical analysis for all experiments was performed using GraphPad Prism version 7.0. Detailed analysis was described in each figure legend. Cell Ranger software pipeline (Version 3.1.0) provided by 10 × Genomics and R package Seurat (Version 3.1.1) were used for scRNA-seq analysis.

For manuscripts utilizing custom algorithms or software that are central to the research but not yet described in published literature, software must be made available to editors and reviewers. We strongly encourage code deposition in a community repository (e.g. GitHub). See the Nature Research guidelines for submitting code & software for further information.

Data

Policy information about availability of data

All manuscripts must include a data availability statement. This statement should provide the following information, where applicable:

- Accession codes, unique identifiers, or web links for publicly available datasets
- A list of figures that have associated raw data
- A description of any restrictions on data availability

Source data are provided with this paper. The scRNA-seq data generated in this study have been deposited in the Gene Expression Omnibus (GEO) under the

accession number GSE167593 (https://www.ncbi.nlm.nih.gov/geo/query/acc.cgi?&acc=GSE167593). All statistical source data and relevant scRNA-seq tables are provided in the Source Data file. Uncropped western blots are provided in the Supplementary Information file.

Field-specific reporting

Please select the one below that is the best fit for your research. If you are not sure, read the appropriate sections before making your selection.

[x] Life sciences [ ] Behavioural & social sciences [ ] Ecological, evolutionary & environmental sciences

For a reference copy of the document with all sections, see nature.com/documents/nr-reporting-summary-flat.pdf

Life sciences study design

All studies must disclose on these points even when the disclosure is negative.

Sample size

No statistical methods were used to pre-determine sample size. The sample size was determined based on similar previous studies of our laboratory[1,2] and on previous experiments using similar methodologies[3,4]. Detailed sample size were described in the figure legends.

References:

[1] Jiang, Lu , et al. "Optogenetic Inhibition of Striatal GABAergic Neuronal Activity Improves Outcomes After Ischemic Brain Injury." Stroke 48.12(2017):STROKEAHA.117.019017.
[2] Zhang, L. Y. , et al. "Microglia exacerbate white matter injury via complement C3/C3aR pathway after hypoperfusion." Theranostics 10.1 (2020):74-90.
[3] Lee, J. H. , et al. "Astrocytes phagocytose adult hippocampal synapses for circuit homeostasis." Nature (2020):1-6.
[4] Chung, W. S. , et al. "Astrocytes mediate synapse elimination through MEGF10 and MERTK pathways." Nature 504.7480(2013):394.

Data exclusions

Animals with which neurological severity score less than 6 at day 1 following stroke, and animals in which histochemical examination showed that the stroke model was unsuccessful were excluded from analysis.

Replication

The experiment for each study was successfully repeated for at least two times.

Randomization

All samples used in the study were randomly allocated into different experimental groups.

Blinding

The behavioral tests, TEM imaging and single-cell RNA sequencing were performed by investigators blinded to the experimental design. For other experiments, investigators were blinded for group allocation, but not blinded for data collection and analysis for practical reasons. We confirmed that all samples were collected and analyzed under the same conditions.

Reporting for specific materials, systems and methods

We require information from authors about some types of materials, experimental systems and methods used in many studies. Here, indicate whether each material, system or method listed is relevant to your study. If you are not sure if a list item applies to your research, read the appropriate section before selecting a response.

Materials & experimental systems

n/a	Involved in the study	
[ ]	[x]	Antibodies
[x]	[ ]	Eukaryotic cell lines
[x]	[ ]	Palaeontology and archaeology
[ ]	[x]	Animals and other organisms
[x]	[ ]	Human research participants
[x]	[ ]	Clinical data
[x]	[ ]	Dual use research of concern

Methods

n/a	Involved in the study	
[x]	[ ]	ChIP-seq
[x]	[ ]	Flow cytometry
[x]	[ ]	MRI-based neuroimaging

Antibodies

Antibodies used

Immunostaining:
Iba-1(1:200, NB100-1028, novusbio, CO), GFAP (1:400, ab53554, Abcam, CA), Synaptophysin (1:200, ab52636, Abcam), Homer-1 (1:200, ab184955, Abcam), MERTK (1:200, ebio-14-5751-82, ebioscience, CA), MEGF10 (1:200, ABC10, Millipore, MA), Mac-2 (1:200, CL8942AP, Cedarlane, Canada), LAMP-2 (1:200, MABC40, Millipore, MA), rat-anti P2RY12 (1:50, 848002, BioLegend), rat-anti F4/80 (1:50, ab6640, Abcam), goat-anti CD31 (1:200, AF3628, R&D), mouse anti MAP2 (1:200, MAB3418, Millipore, MA), rat anti MBP (1:200, ab-7349, Abcam), mouse anti SMI32 (1:200, 801701, BioLegend), Alexa Fluor donkey anti-goat 488, 555, 647 (1:400, A11052, A21432, A21447, Invitrogen, CA), Alexa Fluor donkey anti-rabbit 488 (1:400, A21206, Invitrogen, CA), Alexa Fluor donkey anti-rat 488, 594 (1:400, A21208, A21209, AInvitrogen, CA), Alexa Fluor chicken anti-rat 647 (1:400, A21472, Invitrogen, CA)
Western blot:
MEGF10 (1:700, A10508, ABclonal, MA), MERTK (1:700, AF591, R&D, MN), Synaptophysin (1:800, ab52636, Abcam), Homer-1 (1:800, ab184955, Abcam), β-actin (1:1000, MA5-15739, Invitrogen), HRP-conjugated anti-rabbit or anti-mouse IgG (1:5000, Invitrogen)

IgG staining:
Biotinylated goat anti-mouse IgG (1:50, SP-0022, Bioss, China)

Validation

Antibodies used in our study have been validated by the antibody companies and multiple labs.
The Iba-1 antibody (NB100-1028, novusbio) is reactive to mouse tissue, and has been cited in 175 publications, including Wang J et al., 2019, which used this antibody on mouse brain.
The GFAP antibody (ab53554, Abcam) is reactive to mouse tissue, and has been cited in 156 publications, including Fragola G et al., 2020, which used this antibody on mouse brain.
The Synaptophysin antibody (ab52636, Abcam) is reactive to mouse tissue, and has been cited in 28 publications for both immunostaining and western blot, including Paolicelli RC et al., 2017, which used this antibody on mouse brain.
The Homer-1 antibody (ab184955, Abcam) is reactive to mouse tissue, and has been cited in 5 publications for both immunostaining and western blot.
The MEGF10 antibody (ABC10, Millipore) is reactive to mouse tissue, Morizawa et al.,2017, and Joon-Hyuk Lee et al., 2020, used this antibody on mouse brain.
The MERTK antibody (14-5751-82, ebioscience) is reactive to mouse tissue, and has been cited in 5 publications.
The Mac-2 antibody (CL8942AP, Cedarlane) is reactive to mouse tissue, and has been cited in 39 publications. Katrin et al., 2017 and Irene et al., 2017 used this antibody on mouse adipose tissue.
The LAMP-2 antibody (MABC40, Millipore) is reactive to mouse tissue. In the website, this antibody can be used for immunofluorescence analysis of LAMP-2, including Ferguson et al.,2009, used this antibody on mouse brain.
The MEGF10 antibody (A10508, ABclonal) is reactive to mouse tissue. In the website, expression of MEGF10 was observed in mouse brain for both immunostaining and western blot.
The MERTK antibody (AF591, R&D) is reactive to mouse tissue, and has been cited in 30 publications, including RB Birge et al., 2019, which used this antibody on mouse brain.
The β-actin antibody (MA5-15739, Invitrogen) is reactive to mouse tissue, and has been cited in 190 publications for western blot.
The P2RY12 antibody (848002, BioLegend) is reactive to mouse tissue, and has been cited in 8 publications, including Werneburg S et al., 2020, which used this antibody on mouse brain.
The F4/80 antibody (ab6640, Abcam) is reactive to mouse tissue, and has been cited in 742 publications, including Bautista JA et al., 2021, which used this antibody on mouse brain.
The CD31 antibody (AF3628, R&D) is reactive to mouse tissue, and has been cited in 120 publications, including L Kang et al., 2020, which used this antibody on mouse brain.
The MAP2 antibody (MAB3418, Millipore) is reactive to mouse tissue, and has been cited in 319 publications, including Koh et al., 2015, which used this antibody on mouse brain.
The MBP antibody (ab-7349, Abcam) is reactive to mouse tissue, and has been cited in 187 publications, including Melero et al., 2021, which used this antibody on mouse brain.
The SMI32 antibody (801701, BioLegend) is reactive to mouse tissue, and has been cited in 85 publications, including Kelley et al., 2018, which used this antibody on mouse brain.

Animals and other organisms

Policy information about studies involving animals; ARRIVE guidelines recommended for reporting animal research

Laboratory animals

C57BL/6 mice (male: female=1:1) were purchased from Jackson Laboratory (Stock No. 000664).
MEGF10-flox mice were generated by the trans-NIH Knock-Out Mouse Project (KOMP) with support from the Stanford Transgenic Knockout and Tumor Model Center (TKTC).
MERTK-floxed mice were generated using clustered regularly interspaced short palindromic repeats (CRISPR) technology (Applied StemCell).
Loxp floxed MEGF10 or loxp floxed MERTK mice were crossed with Aldh1l1 Cre-ERT2 or CX3CR1Cre-ERT2 mice (gift from Dr. Won-Suk Chung) and intraperitoneal injected with tamoxifen, to generated specifically gene knockout mice.
All mice with age range 10-12 weeks (male: female=1:1) were used in all experiments.
All mice were kept in a humidity-controlled specific-pathogen fre (SFP) house at 22–25℃ with 12-hrs dark/light cycling, and were allowed to eat and drink freely.

Wild animals

This study did not involve wild animals.

Field-collected samples

This study did not involve samples collected from the fields.

Ethics oversight

Animal studies were reported in accordance with Animal Research: Reporting in Vivo Experiments: ARRIVE guidelines. The procedure for using laboratory animals was approved by the Institutional Animal Care and Use Committee (IACUC) of Shanghai Jiao Tong University, Shanghai, China.

Note that full information on the approval of the study protocol must also be provided in the manuscript.
