# Supplementary material for: Author Correction: Stroke subtype-dependent synapse elimination by reactive gliosis in mice
Source: Nat Commun. 2022 Feb 28;13:1183. doi: 10.1038/s41467-022-28885-6 (PMC8885827; doi:10.1038/s41467-022-28885-6)
Supplement: Supplementary file 1 — Supplementary Figures [file 41467_2022_28885_MOESM1_ESM.pdf]

Supplementary Figure 1

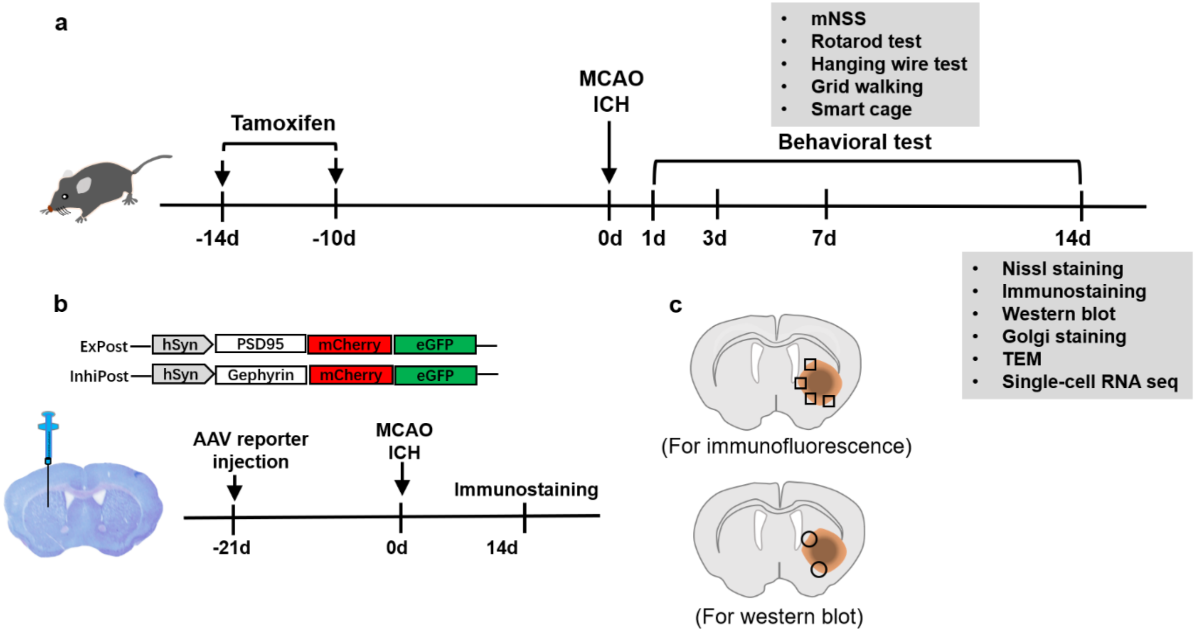

Supplementary Figure 1. Experimental Scheme. (**a**) The schematic illustration of experimental design. (**b**) Illustration of AAV reporter injection. (**c**) Brain regions used for western blot and immunofluorescence analysis.

Supplementary Figure 2

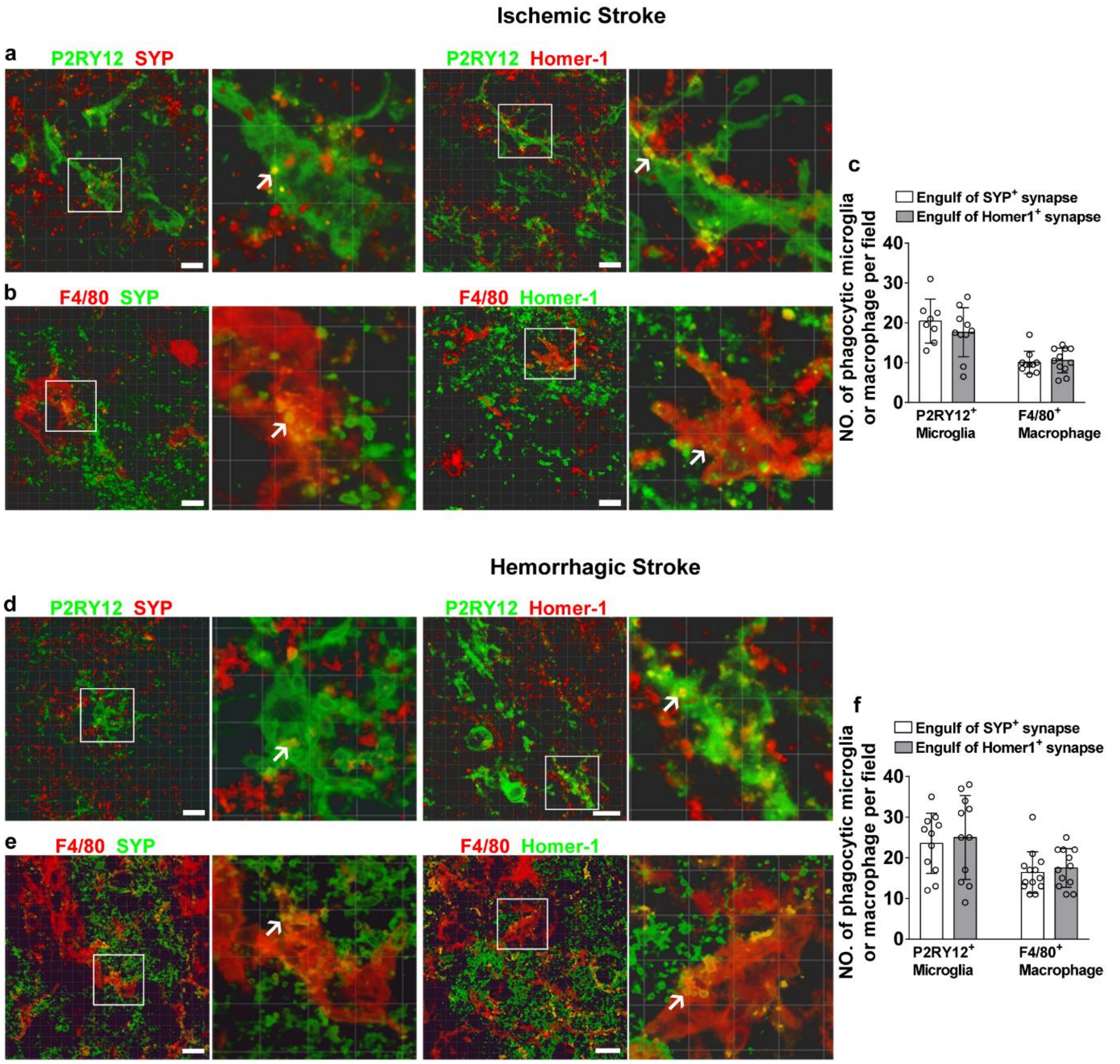

Supplementary Figure 2. Differential contribution of microglia/macrophages in engulfing synapses in ischemic and hemorrhagic stroke. (**a**-**c**) 3D reconstruction images showed SYP$^+$ and Homer-1$^+$ synapse (**a**, red; **b**, green) engulfed by P2RY12$^+$ microglia (green) and F4/80$^+$ macrophages (red) in the ischemic brain, and the proportion of phagocytic cells were quantified. (**d**-**f**) 3D reconstruction images showing SYP$^+$ and Homer-1$^+$ synapse (**a**, red; **b**, green) engulfed by P2RY12$^+$ microglia (green) and F4/80$^+$ macrophages (red) in hemorrhagic brain, and the number of phagocytic cells were quantified. Bar=10 μm. Statistics are derived from 8, 10, 10, 11 slices (**c**) and 11, 11, 13, 12 slices (**f**) (from left to right), n= 3 mice per group. Data are mean ± SD.

Supplementary Figure 3

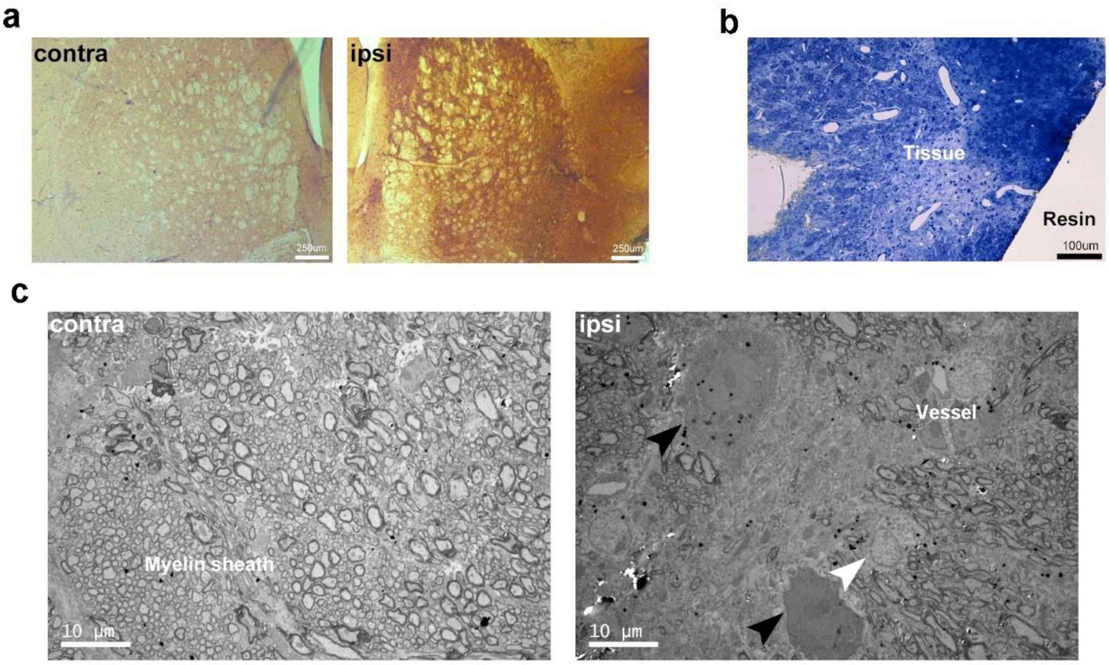

Supplementary Figure 3. Procedure of immuno-TEM. (**a**) DAB staining of glial cells. Left, contralateral striatum; right, ipsilateral striatum. Bar=250 μm. (**b**) Toluidine blue staining of semi-thin sections to distinguish the target tissue. Blue, tissue; white, resin. Bar=100 μm. (**c**) Low magnification of TEM images of striatum. Left, normal striatum, with intact myelin sheath; right, ipsilateral striatum. Arrowheads (black) indicated target glial cells (dark) filled with DAB immunoprecipitation in the cytoplasm and arrowheads (white) indicated unstained cells (light). Bar=10 μm. The experiment was repeated three times independently.

Supplementary Figure 4

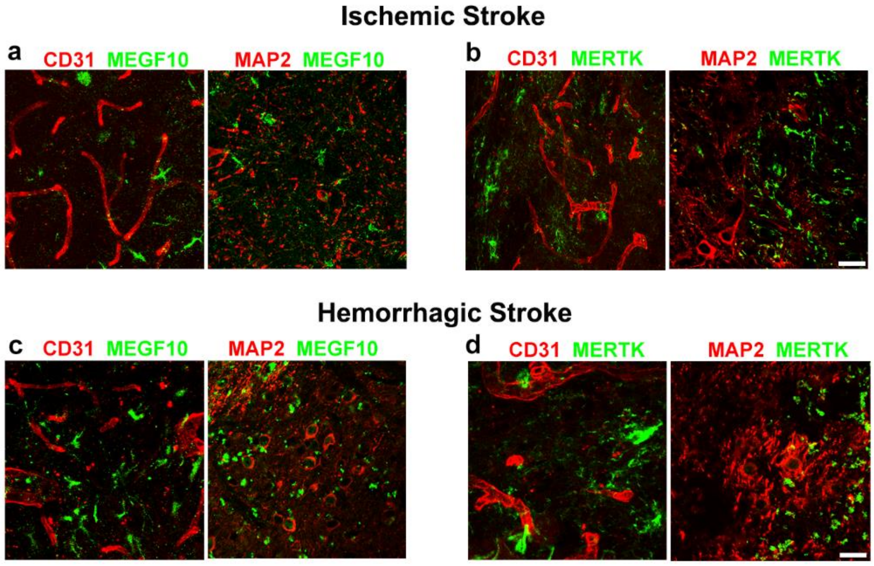

Supplementary Figure 4. **Colocalization of MEGF10/MERTK on other cell types.** MEGF10 and MERTK expression on $MAP2^+$ neurons and $CD31^+$ endothelial cells in both ischemic (**a**, **b**) and hemorrhagic stroke (**c**, **d**). Bar=20 μm. Statistical quantification shown in **Fig. 4**. The experiment was repeated three times independently.

Supplementary Figure 5

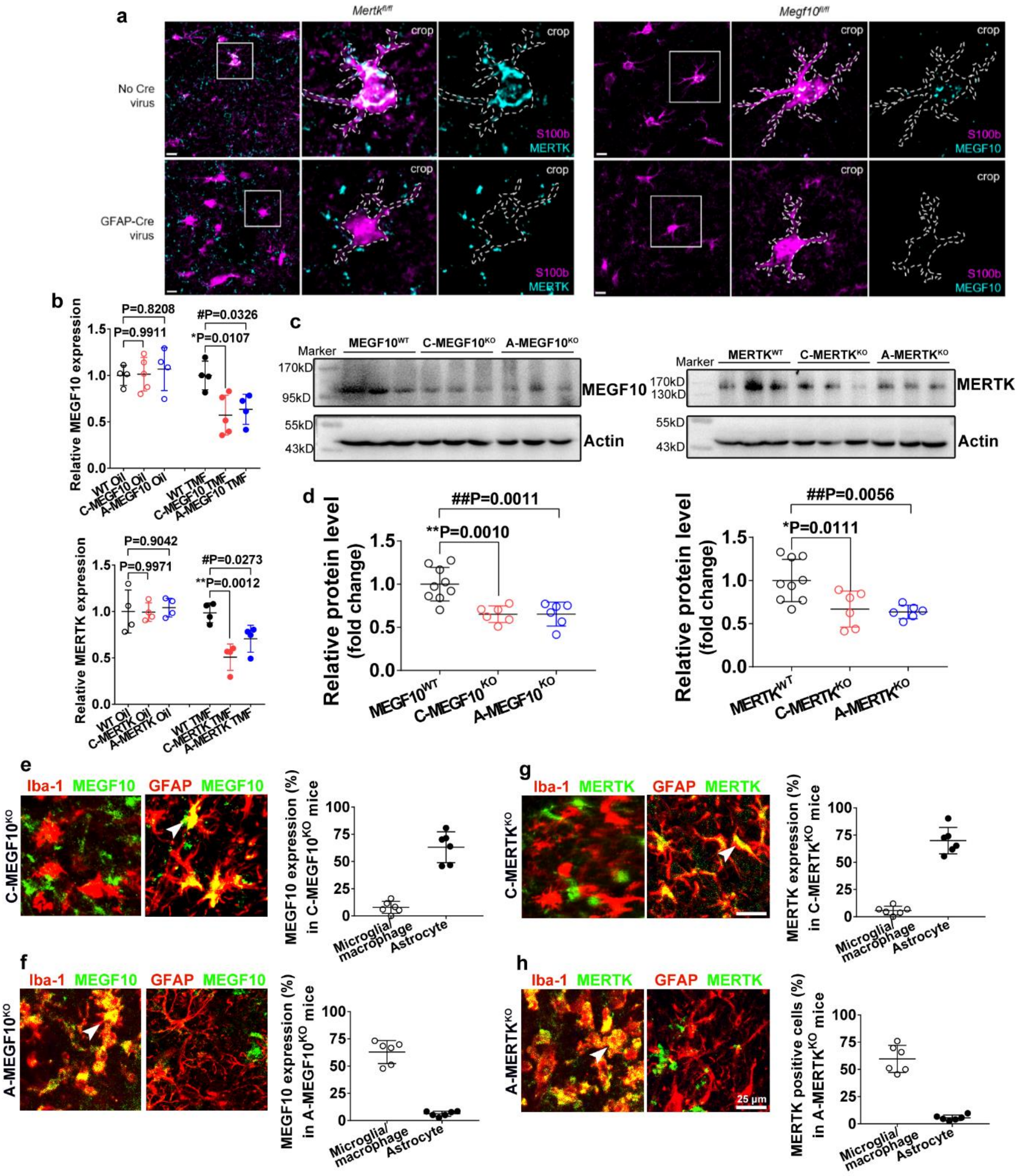

Supplementary Figure 5. Evaluation of MEGF10 and METRK expression in microglia/macrophage- and astrocyte-specific MEGF10 and MERTK knockout mice. (**a**) AAV5-GFAP-Cre virus injection into MEGF10$^{fl/fl}$ or MERTK$^{fl/fl}$ mouse brain showed astrocyte-specific deletion of MERTK and MEGF10 proteins, compared to the control viral vector injected cases. Bar=25 μm. The experiment was repeated twice independently. (**b**) mRNA level of MEGF10 and MERTK in the striatum of oil-injected

WT and transgenic mice, tamoxifen-injected WT and transgenic mice. N=4, 5, 4, 4, 5, 4 mice (up panel) and 4, 4, 4, 4, 4, 4 mice (bottom panel) per group (from left to right). (**c**, **d**) Western blotting and quantification of MEGF10 and MERTK levels in MEGF10^{WT}, C-MEGF10^{KO}, A-MEGF10^{KO}, MERTK^{WT}, C-MERTK^{KO}, A-MERTK^{KO} mice. N=9, 6, 6 mice and 9, 6, 6 mice per group (from left to right). (**e**-**f**) Representative images showed MEGF10 and MERTK were specifically knockout in microglia/macrophages (Iba-1$^+$, red) but not in astrocytes (GFAP$^+$, red) in C-MEGF10^{KO} and C-MERTK^{KO} mice. Arrowheads indicate colocalization of MEGF10 and METRK with astrocytes. Bar=25 μm. (**g**-**h**) Representative images showed MEGF10 and MERTK were specifically knockout in astrocytes but not in microglia/macrophages in A-MEGF10^{KO} and A-MERTK^{KO} mice, as indicated by arrowheads. (**e**-**h**) Statistics are derived from 6 slices, n=3 mice per group. Bar=25 μm. One-way ANOVA followed by Dunnett's test. Data are mean ± SD.

Supplementary Figure 6

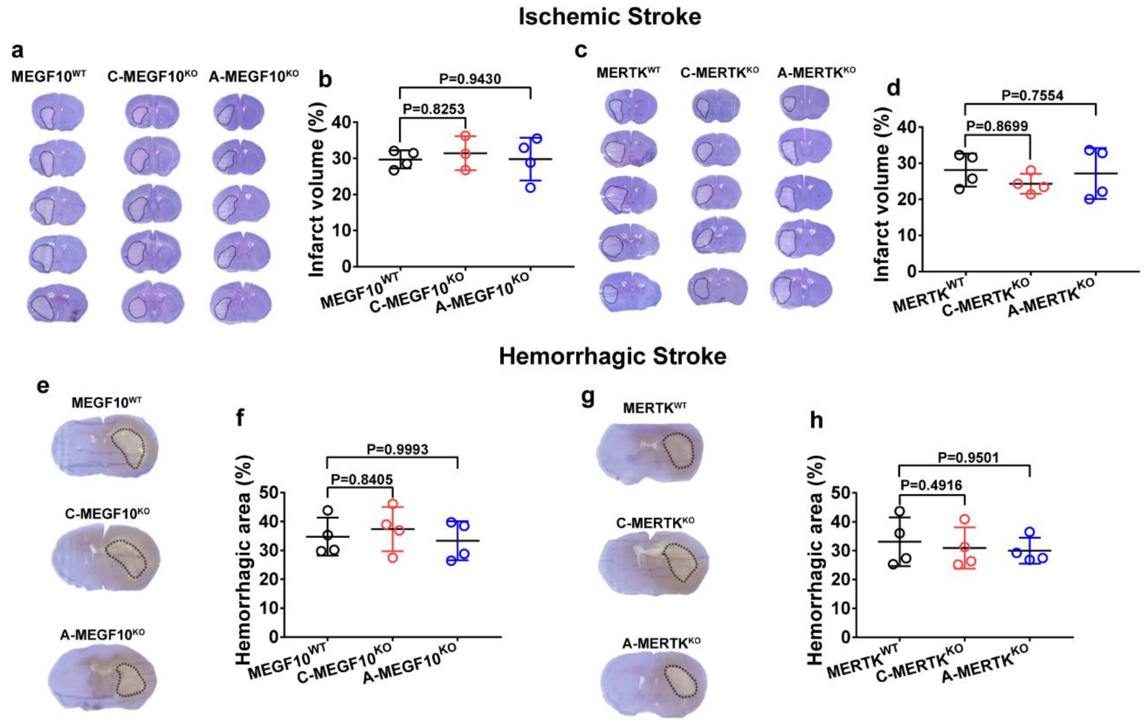

Supplementary Figure 6. Conditional MEGF10 and MERTK knockout did not influence initial brain infarct volume or hemorrhagic area in mice following acute stroke. (a-d) Cresyl violet-stained brain sections and quantification of infarct volume of 1 day following ischemic stroke. N=4, 3, 4 mice (**b**) and 4, 4, 4 mice (**d**) (from left to right). **(e-h)** DAB-stained brain sections and quantification of infarct volume of 1 day post hemorrhagic stroke. Black dashed lines indicated infarct area and core hemorrhagic area of ipsilateral brain. N=4 mice per group. One-way ANOVA followed by Dunnett's test. Data are mean ± SD.

Supplementary Figure 7

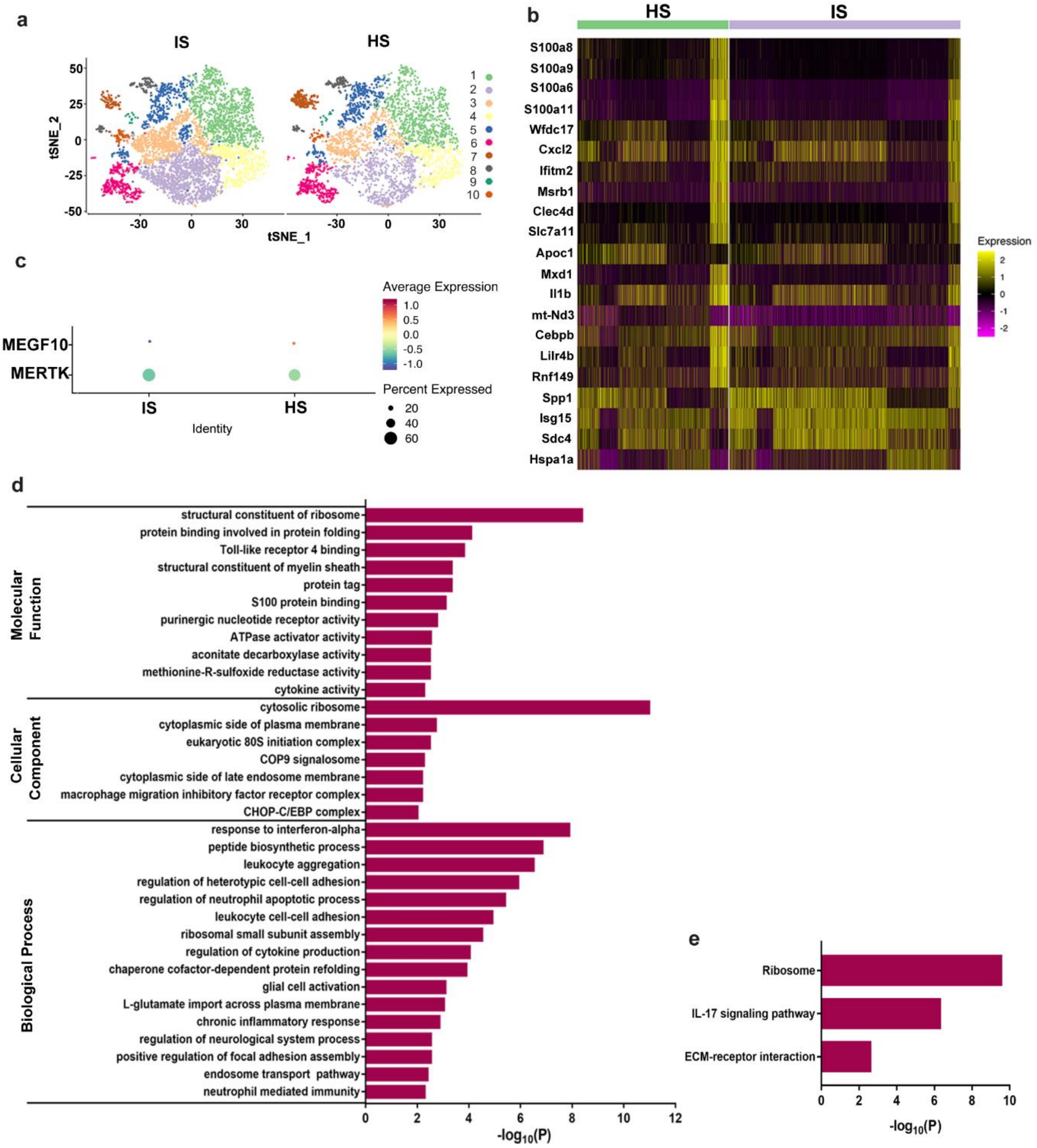

Supplementary Figure 7. scRNA-Seq analysis revealed differentially gene expression of microglia/macrophages in ischemic and hemorrhagic stroke.

(**a**) tSNE map showed subclusters of microglia/macrophages in ischemic stroke (IS) and hemorrhagic stroke (HS). (**b**) Heatmap showed fold change of top genes. (**c**) Dot plot showing the MEGF10 and MERTK expression in microglia/macrophages. (**d-e**) Bar chart showed functional enrichment analysis of differentially expressed genes of microglia between ischemic and hemorrhagic stroke. GO (**d**) and KEGG (**e**) terms were shown in rows and -log10 (*p* value) in columns.

Supplementary Figure 8

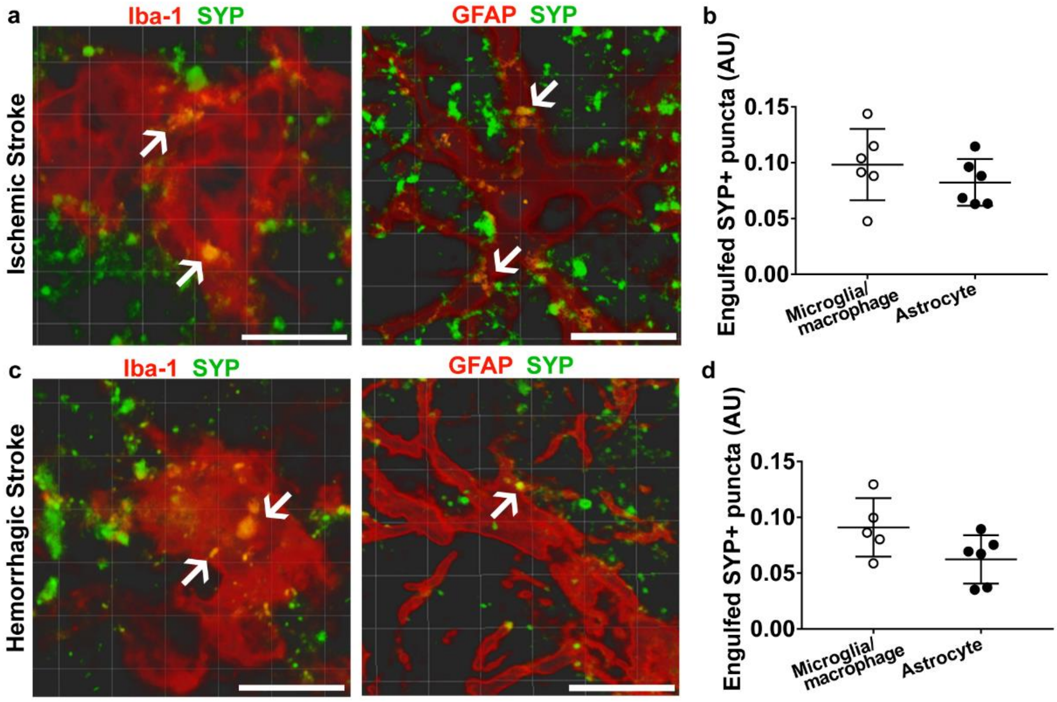

Supplementary Figure 8. **Synapse engulfment by glial cell in acute stroke injury.** SYP^+ synapse (green) engulfed by microglia/macrophages (Iba-1^+, red) and astrocyte ($GFAP^+$, red) at 1 day following ischemic (**a**, **b**) and hemorrhagic stroke (**c**, **d**). Bar=10 μm. AU, arbitrary units. Statistics are derived from 6,6 slices (**b**) and 5, 6 slices (**d**) (from left to right), n=3 mice per group. Data are mean ± SD.

Supplementary Figure 9

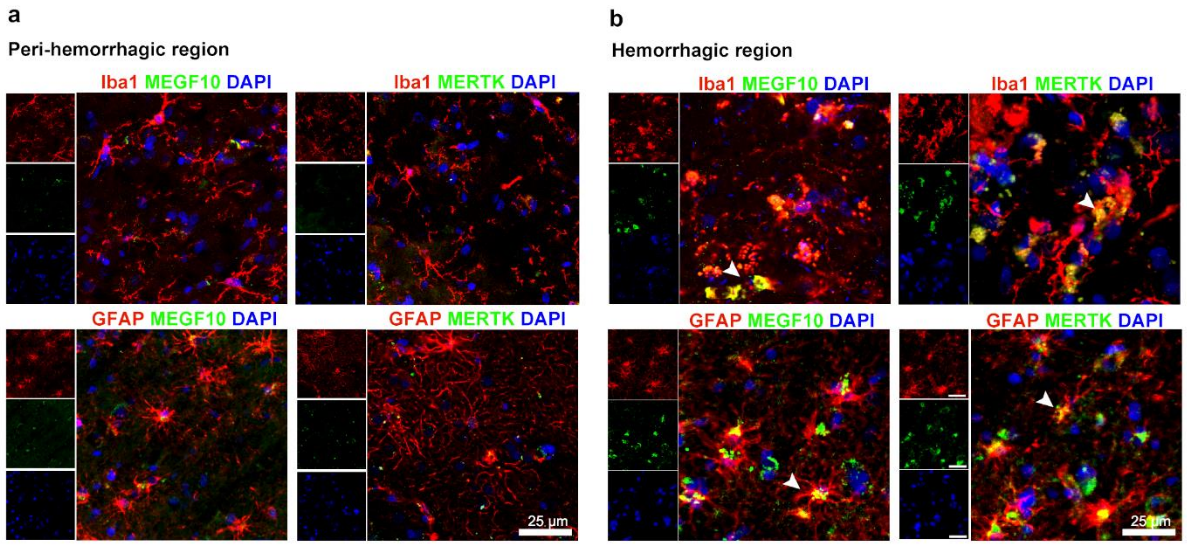

Supplementary Figure 9. MEGF10 and MERTK were expressed in microglia/macrophages and astrocytes in hemorrhagic human brain. Immunostaining results showed colocalization of MEGF10 (green) and MERTK (green) with Iba1$^+$ microglia/macrophages (red) and GFAP$^+$ astrocytes (red) in proximal hemorrhagic region (**a**) and core hemorrhagic region (**b**) of human brain at 12 hrs post hemorrhagic stroke. Bar=25 μm. Arrowheads indicate colocalization. The experiment was repeated twice independently.

Supplementary Figure 10

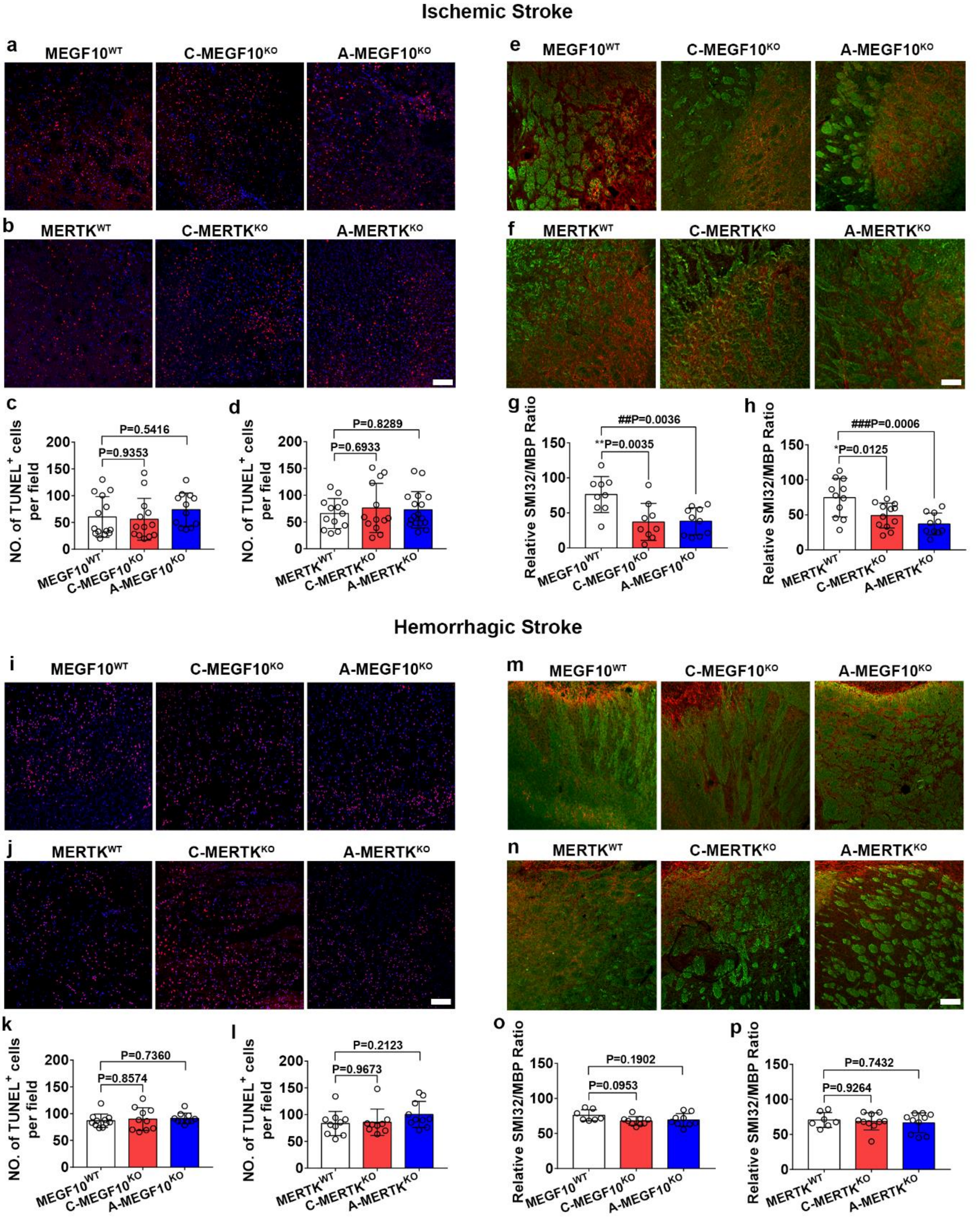

Supplementary Figure 10. The effect of conditional MEGF10 or MERTK knockout on cell death and axonal regeneration in the stroke mouse brain. (**a-d**) Comparing the number of TUNEL⁺ cells of different groups at 1 day following ischemic stroke and quantification. TUNEL, red; DAPI, blue. Statistics are derived from 14, 13, 12 slices (**c**) and 13, 13, 17 slices (**d**) (from left to right), n=4 mice per group. (**e-h**) Co-localization

of MBP (green) and SMI32 (red) indicating axonal degeneration at 14 days following ischemic stroke and quantification. Statistics are derived from 9, 9, 10 slices (**g**) and 11, 12, 10 slices (**h**) (from left to right), n=4 mice per group. (**i**-**l**) Comparing the number of TUNEL$^+$ cells of different groups at 1 day following hemorrhagic stroke and quantification. Statistics are derived from 11, 10, 10 slices (**k**) and 10, 9, 10 slices (**l**) (from left to right), n=4 mice per group. (**m**-**p**) Co-localization of MBP (green) and SMI32 (red) showing axonal degeneration at 14 days post ischemic stroke and quantification. Statistics are derived from 7, 9, 9 slices (**o**) and 7, 10, 10 slices (**p**) (from left to right), n=4 mice per group. Bar=100 μm. One-way ANOVA followed by Dunnett's test. Data are mean ± SD.

Supplementary Figure 11

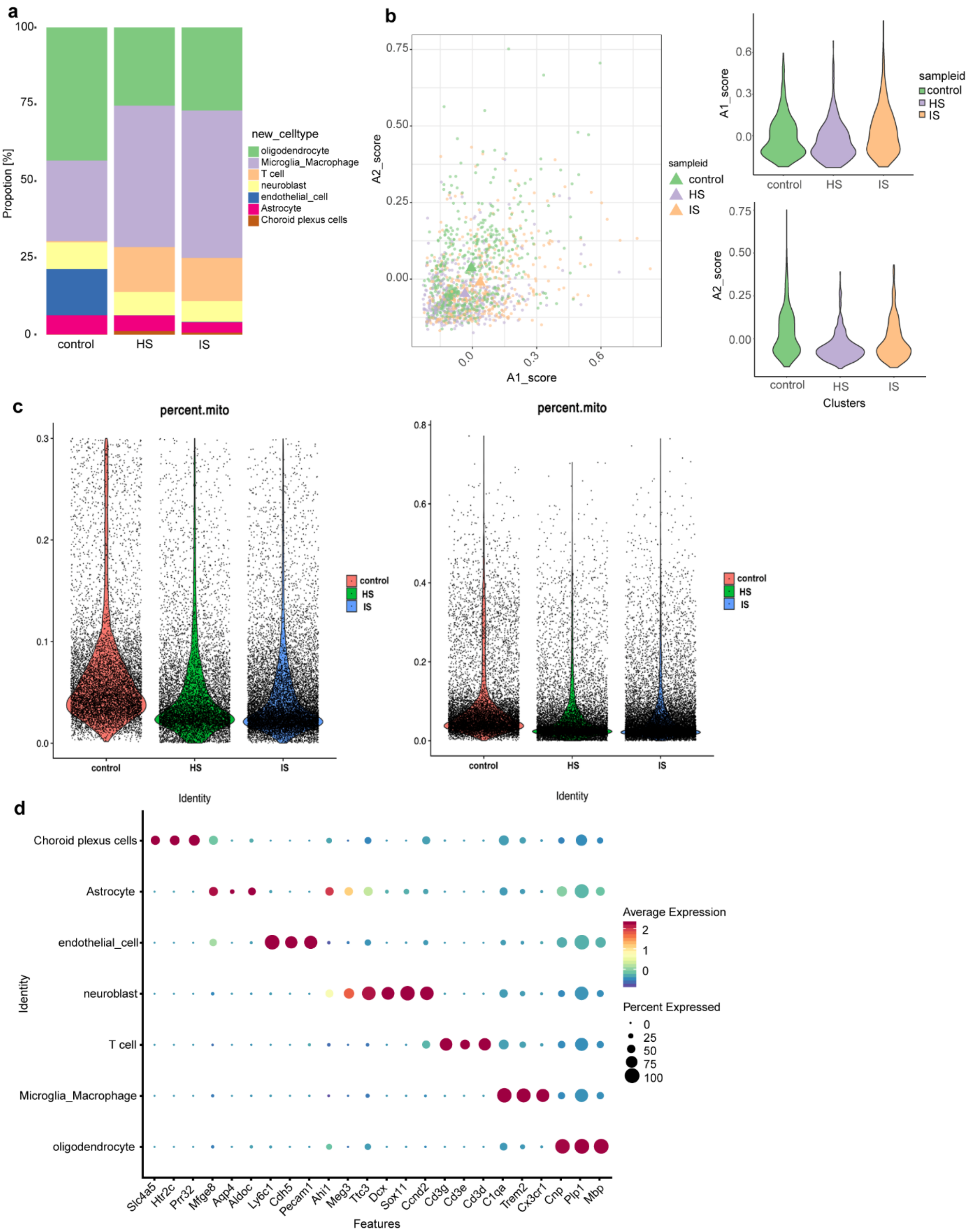

Supplementary Figure 11. (**a**) Histogram showed the proportion of each cell type in each group. (**b**) Cell Plot graph and violin graph showed A1 and A2 scores of astrocytes in healthy control, ischemic, and hemorrhagic mice. (**c**) The number of mitochondrial genes before (left panel) and after quality control (right panel). (**d**)

Marker genes of different cell clusters.

Supplementary Figure 12

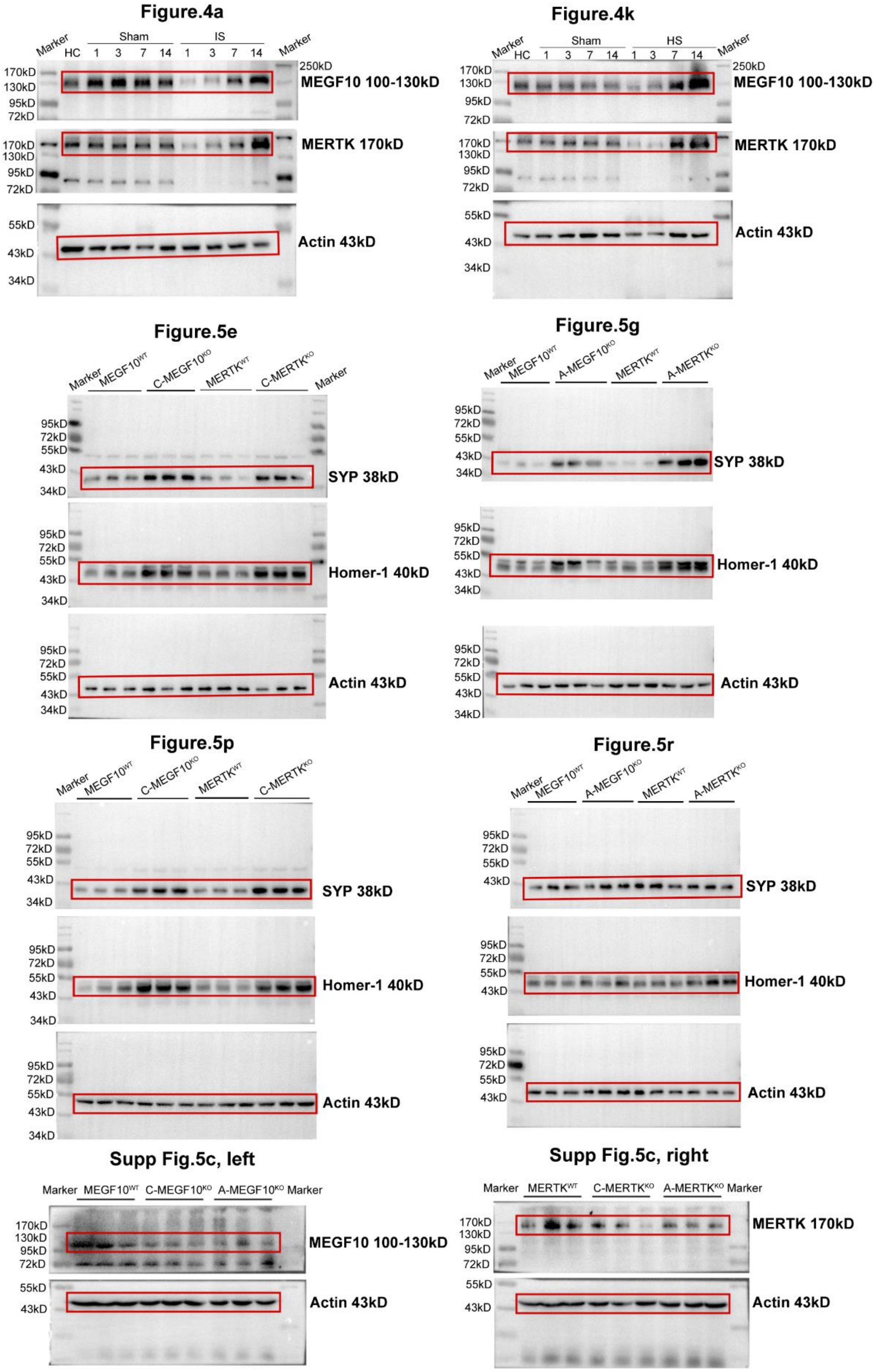

Raw data of western blot
